# Effect of using calcium-silicate and silicone based root canal sealers in bulk or with main core material on bond strength

**DOI:** 10.34172/joddd.2022.036

**Published:** 2022-12-30

**Authors:** Gizem Kadı, Esin Özlek, Yousef Saed

**Affiliations:** ^1^Department of Endodontology, Faculty of Dentistry, Altınbas University, İstanbul, Turkey; ^2^Department of Endodontology, Faculty of Dentistry, Van Yuzuncu Yil University, Van, Turkey

**Keywords:** GuttaFlow Bioseal, iRoot SP, Push-out bond strength, Root canal obturation

## Abstract

**Background.:**

The purpose of this study was to assess the push-out bond strength of calcium-silicate and silicone based root canal sealers in bulk and with main cone.

**Methods.:**

Roots (n=48) randomly divided into 4 groups (n=12) according to the obturation protocol; (1) iRoot SP in bulk; (2) iRoot SP with gutta-percha; (3) GuttaFlow Bioseal in bulk; (4) GuttaFlow Bioseal with gutta-percha. Six horizontal sections were obtained from each root (n=72). Effect of sealers on bond strength was statistically significant (*P*<0.05).

**Results.:**

Highest mean value was obtained in iRoot-Bulk group and lowest in GuttaFlow Bioseal-GP group. Both iRoot SP groups had significantly higher bond strength values than both GuttaFlow Bioseal groups (*P*<0.05). There was no significant difference between iRoot-GP and iRoot-Bulk groups (*P*=0.603) also GuttaFlow Bioseal-GP and GuttaFlow Bioseal-Bulk groups (*P*=0.684).

**Conclusion.:**

Based on findings, using calcium silicate-based sealer in bulk can be also suitable in clinical practice.

## Introduction

The use of bioactive materials that form a mineralized interface has recently increased in popularity. The field of bioceramics was initially root tip repair material, but recently it has started to be a root canal sealer.^1^ Bioceramic root canal sealers are composed of hydraulic calcium silicate and are hydrophilic, insoluble, radiopaque, and aluminum-free materials.^2^ In addition, unlike other root canal sealers, they expand between 0.2%-6% after hardening.^3^

iRoot SP (BioCeramix, Vancouver, Canada) is a bioactive calcium silicate-based root canal sealer that can penetrate dentin tubules, lateral canals, and anatomical deviations due to its small particle size. According to studies, iRoot SP adapts well to dentin walls and provides hermetically sealed root canals.^4,5^ The producer states that iRoot SP root canal sealer can be applicable with gutta-percha cones or that it is also sufficient to use alone. Eymirli et al^5^ reported that using iRoot SP without core material did not cause a difference in dentin tubule penetration, while Nagaş et al^6^ reported that it is stronger in terms of bond strength compared to its use with core material.

GuttaFlow Bioseal (Coltene Whaledent, Langenau, Germany), which recently joined the class of bioactive materials, is a root canal sealer developed as a polydimethylsiloxane-gutta-percha formulation. Due to the calcium silicate in its structure, it exhibits high bond strength with dentin.^7^ Moreover, its viscosity decreases and its fluidity increases under pressure due to its thixotropic feature.^8^ Therefore, it is reported that it penetrates even the lateral canals and consequently, a gap-free root canal filling is obtained.^9,10^

Thermoplastic core materials and root canal sealers fill the root canals. Thermoplastic core materials cannot bond directly to the dentin surface and cannot be used alone because they cannot form a chemical bond. However, with the development of the properties of sealers in recent years, the idea of filling root canals with the “bulk” technique without core material has emerged, which is easier to figure out, and apply more quickly compared to other obturation techniques. When the literature is examined, it is seen that there are few studies evaluating the bond strength of root canals filled with the bulk technique; as far as we know, there is no study on the effects of using GuttaFlow Bioseal root canal sealer with the bulk technique on bond strength.^6,11-13^ Therefore, this study aimed to assess the push-out bond strengths of calcium silicate and silicone-based root canal sealers in bulk or with gutta-percha. The null hypotheses tested were: (*i*) the presence or absence of core material has no effect on push-out bond strength; (*ii*) there is no difference in push-out bond strength between calcium silicate- and silicone-based sealers tested.

## Material and Methods

 Ethical approval for the study was obtained from the Institutional Review Board and the Ethics Committee of the University, on 21/01/2022 presenting with statement number 2022/01-14. In this study, 48 single-canal, closed apex, straight rooted and non-carious mandibular premolar teeth extracted for periodontal or orthodontic reasons were used. The mesiodistal and bucco-palatal radiographs of the teeth were taken and it was checked whether they had a single root canal. The calculus and periodontal ligaments were cleaned using periodontal curettes and all teeth were kept in distilled water until use.

###  Specimen preparation

 The teeth were sectioned off with a slow speed diamond saw (IsoMet, Buehler, Lake Bluff, IL, USA) under water coolant and their crowns were removed for adjusting the root length in standardized of 12 mm. 15 K-files (MicroMega, Besancon, France) were inserted into the canal and the working length was established to be 1 mm shorter than the apical foramen and recorded. The chemomechanical preparation was applied with the Protaper Next (Dentsply, Maillefer, Ballaigues, Switzerland) rotary file system up to X3. At each file change, the root canals were irrigated with 2 mL of 5.25% NaOCl (Microvem AF, İstanbul, Turkey) using a 31G side-ventilated needle. Apical patency was checked by passing a 10 K-file through the apex. After root canal preparation, 5 mL each of 17% EDTA (Imicryl, Konya, Turkey) and 5.25% NaOCl were used for 1 minute for final irrigation followed by rinsed with 5 mL of distilled water for eliminate the irrigants residues.

 Afterwards all samples were divided into 4 groups (n = 12/group) according to the root canal filling protocol; (1) iRoot SP (BioCeramix, Vancouver, Canada) in bulk; (2) iRoot SP with gutta-percha; (3) GuttaFlow Bioseal (Coltene Whaledent, Langenau, Germany) in bulk; (4) GuttaFlow Bioseal with gutta-percha. All root canal sealers were applied following the manufacturer’s recommendations. The canals in groups 1 and 3 were filled with sealer only. The filling of the root canals in groups 2 and 4 was performed with a single cone technique with sealer and size 30, 0.06 taper gutta-percha point (Dentsply Maillefer). After the access cavities were covered with temporary filling material (Coltosol F; Coltene/Whaledent AG), all specimens were kept at 37°C and 100% humidity for 2 weeks for the root canal sealers to set.

###  Push-out bond strength test

 All roots were cut 3, 6, and 9 mm horizontally from the apical region with a low-speed diamond saw (Isomet, Buehler, Lake Bluff, IL, USA) under water cooling. Six sections of 1 ± 0.2 mm thickness were obtained from each tooth. Section thicknesses were confirmed with a digital caliper. Samples with an oval root canal structure (i.e., filling with voids) were excluded from the study and instead, a new sample prepared according to the current protocol applicable to that group was added.

 Stainless steel plungers which almost completely covered the main cone, which did not contact the canal wall were used. In the push-out test, custom-made plungers of 1.10, 0.8 and 0.3 mm were used for the coronal, middle and apical regions, respectively. An apical to coronal force was applied by plungers operating at a crosshead speed of 1 mm/min attached to the test machine (Shimadzu Corporation, Kyoto, Japan). The force causing bond failure was recorded as Newton by Trapezium X Software (Shimadzu Corporation, Kyoto, Japan) then the following formula is used to convert to megapascals (Mpa).

Push-out bond strength (MPa) = N/A; N = maximum failure load, A = adhesion area (mm^2^). Calculation of the bonding surface area of each slices was done using the following formula: [π (r1 + r2)] x [(r1 − r2)2 + h2 ]1/2, where π = 3.14, r1 = smaller radii, r2 = larger radii, and h = the thickness of the section in mm^3^.^14^

###  Analysis of failure modes

Failure mode examination was performed with a stereomicroscope (Leica M320 F12, Leica Microsystems, Wetzlar, Germany) under 40x magnification. Both sides of the samples were examined and failure modes were determined in accordance with specified classification: (*i*) adhesive failure (dentin/sealer interface), (*ii*) cohesive failure (both dentin/sealer and sealer/main cone interface) and (*iii*) mixed failure (both adhesive and cohesive).

###  Statistical evaluation

The statistical analysis of the obtained data was performed with the IBM Statistical Package for the Social Sciences (SPSS) v23 software package. Analysis of the data using Shapiro-Wilk tests showed that the data were not normally distributed. Therefore, the differences between bond strength values were compared non-parametrically Kruskal-Wallis test. Mann-Whitney U test was used for pairwise comparison of the groups. The results were evaluated at a significance level of *P* < 0.05.

## Results

###  Bond strength

The highest mean value was obtained in the iRoot-Bulk group (6.60 ± 4.80) and the lowest in the GuttaFlow Bioseal-GP group (3.38 ± 2.98). The effect of the root canal sealer on the bond strength was statistically significant (*P* < 0.05) (Table 1). In addition, Mann Whitney U analysis showed that the iRoot-GP group had higher bond strength values than the GuttaFlow Bioseal- GP and GuttaFlow Bioseal-Bulk groups, and the difference was statistically significant (*P* < 0.05). The iRoot-Bulk group showed higher binding values than the GuttaFlow Bioseal-GP and GuttaFlow Bioseal-Bulk groups, and this difference was statistically significant (*P* < 0.05). However, the difference between the iRoot-GP and iRoot-Bulk groups and between the GuttaFlow Bioseal-GP and GuttaFlow Bioseal-Bulk groups was not statistically significant (*P* > 0.05). There was no significant difference between iRoot-GP and iRoot-Bulk group (*P* = 0.603). Also, there was no significant difference between GuttaFlow Bioseal-GP and GuttaFlow Bioseal-Bulk group (*P* = 0.684) (Table 2).

**Table 1:** Descriptive statistics of push-out bond strenght values

**Push-out bond strenght (MPa)**	**Mean±SD**	**Max**	**Min**	***P*** **value** ^a^
GuttaFlow-GP	3.38 ± 2.98	10.52	0.75	0.000
GuttaFlow-Bulk	3.67 ± 3.43	14.19	0.78	
iRoot-Bulk	6.60 ± 4.80	20.45	1.19	
iRoot-GP	5.89 ± 3.70	16.96	1.32	

SD: Standart deviation, Max: Maximum, Min: Minimum. ^a^Kruskal-Wallis Test, *P < 0.05.

**Table 2:** Mann-Whitney U test for intergroup comprasion

**Groups**	**GuttaFlow-GP**	**GuttaFlow-Bulk**	**iRoot-Bulk**	**iRoot-GP**
GuttaFlow-GP	-	0.684	0.000^*^	0.000^*^
GuttaFlow-Bulk	0.684	-	0.000^*^	0.000^*^
iRoot-Bulk	0.000^*^	0.000^*^	-	0.603^*^
iRoot-GP	0.000^*^	0.000^*^	0.603	-

*P < 0.05 significant.

###  Failure modes

**Figure 1:**
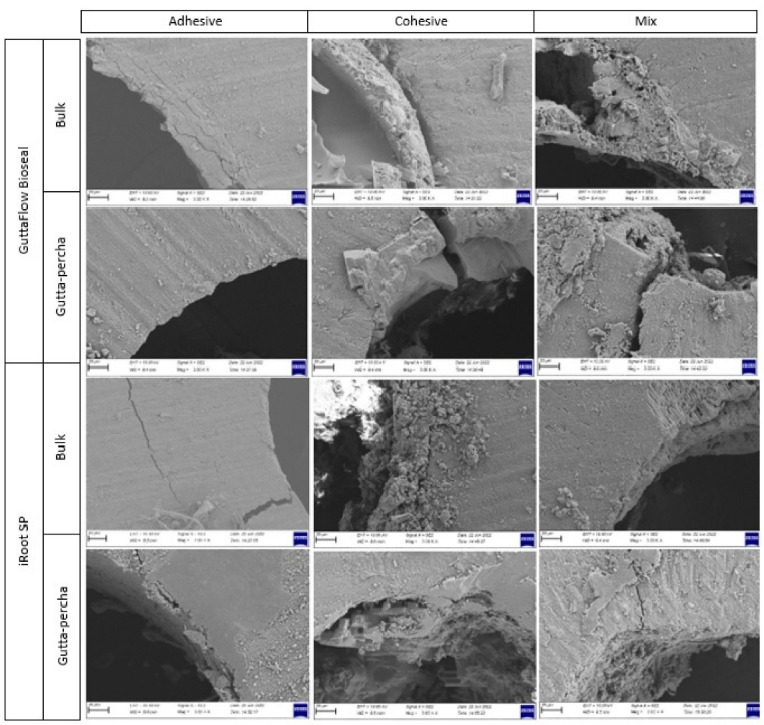
Representative scanning electron microscope images showing failure modes after bond failure.

The failure modes according to the analysis are presented in Table 3 for each group. The dominant failure mode in GuttaFlow Bioseal-Bulk, iRoot-GP and iRoot-Bulk groups, was adhesive (between sealer and dentin). The failure mode in the GuttaFlow Bioseal-GP group was mostly seen in the cohesive type. Figure 1 shows scanning electron microscopy images of the coronal sections of each group, representing adhesive, cohesive and mix failure modes.

**Table 3:** Failure modes (%) for each group

	**GuttaFlow-GP**	**GuttaFlow-Bulk**	**iRoot-Bulk**	**iRoot-GP**
Adhesive	25	75	52.7	51.3
Cohesive	62.5	12.5	26.3	37.5
Mixed	12.5	12.5	20.8	11.1

## Discussion

Root canal sealers penetrate irregularities in the root canal system and form an incomparable nexus between the root surface and obturation material. Therefore, root canal sealers are needed in root canal filling regardless of the filling technique.^15,16^ This study investigated the effect of using two bioactive root canal sealers and the main core material in combination on bond strength. The results reveal that the calcium silicate-based sealer, when used in bulk or with the main cone, presented remarkably higher bond strength values than both the silicone-based sealer in bulk and the experimental version used with the main cone (*P* < 0.05); however, the use of core material had no remarkable difference on bond strength (*P* > 0.05). Therefore, the null hypothesis was partially rejected.

When the root canal sealer is used with the core material, the sealer exists as a superficial film layer and forms two interfaces dentin/sealer and sealer/main cone.^17^ This leads to a reduction in the homogeneity of the filling and a deterioration of the obturation quality due to the gap between the sealer and the core. When using a root canal sealer in bulk, one interface is formed between the obturation material and the root surface. This way, an ideal monoblock structure is obtained throughout the root canal system. Also, by eliminating the dislocation resistance limited by the main cone, ideal sealer/dentin dislocation resistance is provided.^6^ This study reports the average bond strength values as iRoot-Bulk, iRoot-GP, GuttaFlow Bioseal-Bulk, and GuttaFlow Bioseal-GP, respectively. The results support the findings of previous studies using the same or a differentiated push-out test, which stated that root canal sealers in bulk form resulted in higher bond strength values compared to the use of core material.^6,11,13^

The C-factor is one factor that influences the strong bond of root canal sealers with the root surface. The bonded to the unbonded surface ratio is referred to as the configuration factor or C-factor. During polymerization, the unbound surface moves to compensate for shrinkage stress. When the unbound surface becomes smaller, such as in a long and narrow root canal, the shrinkage stress cannot be adequately compensated, and the likelihood of the root filling detaching from the dentin surface increases. As a result, it has been reported that the C-factor in root canals negatively correlates with sealer thickness, and the C-factor decreases when root canals are filled with sealer only.^18^ This explains why the iRoot SP bulk group had a higher bond strength than the iRoot SP-GP group and the GuttaFlow Bioseal-Bulk group had a higher bond strength than the GuttaFlow Bioseal-GP group, although there was no statistically significant difference. Another reason could be that when the root canals are filled with sealer alone, the calcium ions released are higher, and biomineralization increases. Retana-Lobo et al^13^ reported that BioRoot^TM^ RCS had a higher bond strength when used without gutta-percha and related this result to the fact that bioactive sealers, when used alone, preserve their bioactivity for a long time and show a high amount of calcium ion release.

Thanks to the calcium silicate in their structure, bioactive root canal sealers form apatite interface deposits upon contact with liquids, which form a physical bond with the dentin surface and thus exhibit high bond strength with dentin.^7^ At the same time, it penetrates deeper into the dentinal tubules thanks to its small particle size and viscosity,^19^ presumably increasing the adhesion efficiency to root canal dentin.^6^ Hoikkala et al reported that GuttaFlow Bioseal contains bioactive glass-ceramic particles embedded in a polydimethylsiloxane matrix of 20-40 µm.^20^ In another study, GuttaFlow Bioseal was reported to have a particle size of about 10 μm.^21^ Akcay et al also concluded that iRoot SP is a root canal sealer with a very small particle size ( < 2 μm) and thus has a significantly larger dentin-tubule penetration area than GuttaFlow Bioseal.^22^ According to the given information, we consider it a predictable result that the bond of the root canal sealer to the dentin increases with decreasing particle size, as in this study, and that the iRoot SP groups have a higher bond strength than the GuttaFlow Bioseal groups.

In this study, the push-out test, a widely accepted method, was used to evaluate the bond strength of root-filling materials at the root-dentin interface.^13,23^ In addition to being a relatively simple method compared to tensile and shear strength tests, the push-out test can replicate similar clinical conditions and has low technical sensitivity,^24,25^ and more homogeneous stress distribution at the dentin interface has been reported compared to other methods.^26^ Another advantage of this method is that multiple sections can be obtained from a single root, and thus the bond strength of the filling material at the dentin interface can be measured on all surfaces of the root canal.^27^ Since the evaluation of 1 mm thick specimens in the bond strength test is considered reliable, 1 ± 0.2 mm thick sections were included in this study.^28^

The results show that the predominant failure mode of iRoot SP sealer is adhesive when used with both gutta-percha and bulk. Due to the nature of calcium silicate-based root canal sealer, it is reported that the failure mode is usually adhesive, as adhesion with the bulk core material is limited or incomplete.^6^ The results are consistent with other studies reporting that calcium silicate-based sealers adhere much more strongly to dentin than to the main core material.^6,11^ In the GuttaFlow Bioseal-GP group, which was applied as a thin film, failure was cohesive within the sealer itself, whereas in the GuttaFlow Bioseal-Bulk group, which was applied as a thicker film layer, adhesive failure was observed between the dentin and the sealer. We believe that this difference is related to the fact that plastic deformation of the gutta-percha cone negatively affects the push-out bond strength.^13^

## Conclusion

 Within the limitations of this in vitro study, it can be concluded that the group that presented the highest dislocation resistance to radicular dentin was the calcium silicate-based sealer in the bulk group, and the silicone-based sealer with a gutta-percha group presented the lowest mean bond strength values. When the sealer is available as a thin layer or bulk-filled, it behaves differently in terms of bond strength. However, obturation quality depends on bond strength and sealer properties, such as sealing ability and voids in filling. Further studies are needed to make clinical recommendations for using bioactive sealers in bulk as an obturation technique.

## Funding

 The authors declared that this study has received no funding.

## Ethics Approval

 Ethics committee approval was received for this study from the ethics committee of Van Yüzüncü Yıl University with statement number 2022/01-14.

## Competing Interests

 The authors declared that they have no conflicts of interest related to this study.
